# Therapeutic Response Monitoring with ^89^Zr-DFO-Pertuzumab in HER2-Positive and Trastuzumab-Resistant Breast Cancer Models

**DOI:** 10.3390/pharmaceutics14071338

**Published:** 2022-06-24

**Authors:** Minwoo Kang, Jong Il Shin, Sangjin Han, Jung Young Kim, Jeonghoon Park, Kwang Il Kim, Joo Hyun Kang, Tae Sup Lee

**Affiliations:** 1 Division of RI Application, Korea Institute of Radiological and Medical Sciences (KIRAMS), 75 Nowon-ro, Nowon-gu, Seoul 01812, Korea; mwkang@kirams.re.kr (M.K.); shinjongil00@gmail.com (J.I.S.); sjplussin@naver.com (S.H.); jykim@kirams.re.kr (J.Y.K.); kikim@kirams.re.kr (K.I.K.); kang2325@kirams.re.kr (J.H.K.); 2 Korea Atomic Energy Research Institute (KAERI), 29 Geumgu-gil, Jeongeup-si 56212, Korea; parkjh@kaeri.re.kr

**Keywords:** breast cancer, HER2, trastuzumab, pertuzumab, heat shock protein 90 (HSP90), 17-DMAG, Zirconium-89, positron emission tomography (PET)

## Abstract

Immuno-positron emission tomography (PET) has great potential to evaluate the target expression level and therapeutic response for targeted cancer therapy. Immuno-PET imaging with pertuzumab, due to specific recognition in different binding sites of HER2, could be useful for the determination of the therapeutic efficacy of HER2-targeted therapy, trastuzumab, and heat shock protein 90 (HSP90) inhibitor, in HER2-expressing breast cancer. The aim of this study is to evaluate the feasibility of monitoring therapeutic response with ^89^Zr-DFO-pertuzumab for the treatment of HER2-targeted therapeutics, trastuzumab, or the HSP90 inhibitor 17-DMAG, in trastuzumab-resistant JIMT-1 breast cancer models. We prepared an immuno-PET imaging agent using desferoxamine (DFO)-pertuzumab labeled with ^89^Zr and performed the biodistribution and PET imaging in breast cancer xenograft models for monitoring therapeutic response to HER2-targeted therapy. ^89^Zr-DFO-pertuzumab was successfully prepared and showed specific binding to HER2 in vitro and clearly visualized HER2 expressing JIMT-1 tumors. ^89^Zr-DFO-pertuzumab had prominent tumor uptake in HER2 expressing JIMT-1 tumors. JIMT-1 tumors showed trastuzumab-resistant and HSP90 inhibitor sensitive characterization. In immuno-PET imaging, isotype antibody-treated JIMT-1 tumors had similar uptake in trastuzumab-treated JIMT-1 tumors, but 17-DMAG-treated JIMT-1 tumors showed greatly reduced uptake compared to vehicle-treated tumors. Additionally, HER2 downregulation evaluated by immuno-PET imaging was verified by western blot analysis and immunofluorescence staining which resulted in a significant reduction in the tumor’s HER2 level in 17-DMAG-treated JIMT-1 tumors. ^89^Zr-DFO-pertuzumab immuno-PET may be clinically translated to select pertinent patients for HER2-targeted therapy and to monitor the therapeutic response in HER2-positive cancer patients under various HER2-targeted therapeutics treatments.

## 1. Introduction

Immuno-positron emission tomography (PET), the combination of PET with monoclonal antibodies (mAbs), is an attractive approach to improve the diagnostic characterization of various diseases, combining PET system and mAbs with high sensitivity and specificity, respectively [1]. Immuno-PET imaging allows the visualization and quantification of target expression at the whole-body level and could be used to predict the efficacy and toxicity of antibody-based therapeutics, mAbs, radioimmunotherapeutic agents, and antibody-drug conjugates (ADCs), as well as to select individual patients or targets and determine the dosing schedule of targeted therapeutics [2,3,4,5]. ^89^Zr (78.4 h) has optimal physical half-life for the distribution of mAbs in vivo and ^89^Zr-based immuno-PET imaging has been applied in not only academia but also pharma industry [6].

Human epidermal growth factor receptor 2 (HER2, ErbB2) is a member of the ErbB family of receptor tyrosine kinases (RTKs), which includes EGFR (ErbB1), HER3 (ErbB3), and HER4 (ErbB4). HER2 overexpression results in the autophosphorylation of tyrosine residues by dimerization and initiates a variety of signal transduction pathways leading to cellular proliferation and tumorigenesis [7]. HER2 expression occurs in approximately 20% of human breast cancer. HER2-targeted therapies, trastuzumab (Herceptin^®^) and/or pertuzumab (Perjeta^®^) and/or chemotherapeutic agents, are effective therapeutic regimens in breast cancer with HER2 overexpression and/or amplification. Recently, the treatment of breast cancer has adopted a multidisciplinary approach that includes local treatments with surgery and radiation and systemic therapies with chemotherapy, hormone therapy, poly (ADP-ribose) polymerase (PARP) inhibitors, and immunotherapy such as immune checkpoint inhibitors depending on the cancer subtype and disease stage [8,9]. Additionally, antibody-drug conjugates, Ado-trastuzumab emtansine (Kadcyla^®^) and trastuzumab deruxtecan (Enhertu^®^), have been approved by the FDA for the treatment of advanced HER2-positive breast tumors [10,11,12]. Heat shock protein 90 (HSP90) inhibitors have been developed and showed therapeutic efficacy in animal models, and their safety and efficacy were evaluated in HER2-positive cancer patients [13,14].

Heat shock protein 90 (HSP90) has emerged as a promising target for cancer therapy [15]. HER2 is dependent upon HSP90 for its stability throughout the whole life span of the receptor, including the maturation process in the ER, and during the residency of the receptor at the plasma membrane [16,17]. Consequently, the degradation of HER2 upon inactivation of HSP90 occurs from both the ER and the plasma membrane [18]. In particular, the HSP90 inhibitor, 17-(allylamino)-17-demethoxygeldanamycin (17-AAG), showed therapeutic efficacy in trastuzumab-resistant breast cancer animal models [19] and the HSP90 inhibitor, 17-AAG, combined with trastuzumab also had antitumor activity in trastuzumab refractory HER2 overexpressing breast cancer patients [20]. Since HER2 is a key client protein of HSP90, analyzing HER2 expression status using immuno-PET imaging at the whole-body level would be useful for the evaluation of therapeutic efficacy by HSP90 inhibitors.

Pertuzumab, one of the anti-human epidermal growth factor receptor-2 (HER2) recombinant humanized mAbs, binds to the extracellular domain II of HER2 which is a different epitope from that of trastuzumab (domain IV) and prevents the formation of the HER2 heterodimerization [21]. Although pertuzumab has demonstrated some activity in patients with HER2-positive breast cancer that progressed during therapy with trastuzumab, the combination of pertuzumab and trastuzumab seems to be more active than monotherapy [22]. Therefore, pertuzumab as an immuno-PET imaging agent could be used to evaluate in vivo HER2 expression and the pharmacodynamics of HER2 by various HER2-targeted therapeutic agents.

Immuno-PET imaging provides us with the ability to visualize and quantify HER2 expression and evaluate therapeutic response by HER2-targeted therapy. HER2-based PET tracers including ^18^F-HER2 aptamer [23], ^68^Ga- [24], and ^64^Cu-HER2 affibodies [25], ^89^Zr-trastuzumab [26], and ^89^Zr-pertuzumab [27,28] were applied for HER2-positive tumor detection in animal models and clinical studies. In addition, the non-invasive PET imaging method utilizing ^64^Cu-trastuzumab [29], ^89^Zr-trastuzumab [30], and ^89^Zr-pertuzumab [31] has been used to monitor response for HER2-targeted therapies.

In this study, we prepared an immuno-PET imaging agent using desferoxamine (DFO)-pertuzumab labeled with ^89^Zr, performed the biodistribution and PET imaging in breast cancer xenograft models for monitoring therapeutic responses to HER2-targeted therapy, and evaluated the usefulness of ^89^Zr-DFO-pertuzumab for the treatment of HER2-targeted therapeutics, trastuzumab or HSP90 inhibitor, 17-dimethylamino- ethylamino-17-demethoxygeldanamycin (17-DMAG), in trastuzumab-resistant JIMT-1 breast cancer models.

## 2. Materials and Methods

### 2.1. Cell Culture

Breast cancer cell line, MDA-MB-231 was obtained from the American Type Culture Collection (ATCC, HTB-26, Manassas, VA, USA) and JIMT-1 was purchased by AddexBio (C0006005, San Diego, CA, USA). Cell lines were grown in a DMEM culture medium supplemented with 10% fetal bovine serum (FBS) and 1% antibiotics/antimycotics (Gibco, 15240096, Waltham, MA, USA, penicillin 10,000 units/mL, streptomycin 10,000 μg/mL, amphotericin B 25 μg/mL). The cells were kept in humidified 95% air and 5% CO_2_ at 37 °C.

### 2.2. Flow Cytometry

Breast cancer cells were harvested and washed with PBS containing 1% (*w*/*v*) bovine serum albumin (Sigma Aldrich, Saint Louis, MO, USA). Cells were incubated with 10 μg of pertuzumab (Perjeta^®^, Roche, Basel, Switzerland) or isotype antibody, rituximab (Mabthera^®^, Roche, Basel, Switzerland) for 1 h at 4 °C. After washing, 1 μL of monoclonal anti-human FITC-conjugated IgG antibody (Sigma Aldrich, F5016, Saint Louis, MO, USA) was added and incubated for 1 h at 4 °C. Stained cells were analyzed using FACS Calibur and CellQuest software (BD Biosciences Immunocytometry System, San Jose, CA, USA) to measure the HER2 expression level at the cell surface.

### 2.3. Preparation and Characterization of ^89^Zr-DFO-Pertuzumab

Anti-HER2 antibody, pertuzumab, was buffer-exchanged with 0.1 M sodium bicarbonate buffer, pH 8.5, and concentrated to 10 mg/mL using Vivaspin-20 centrifugation tubes with 50 kDa MW cutoff (Sartorius, Hannover, Germany). Pertuzumab reacted with 10 equivalents of p-SCN-Bn-deferoxamine (p-SCN-Bn-DFO, Macrocyclics, Plano, TX, USA) dissolved in dimethyl sulfoxide. Conjugation was allowed to proceed at room temperature for 2 h with stirring and continued at 4 °C overnight. DFO-pertuzumab was finally concentrated to 2 mg/mL in 10 mM 4-(2-Hydroxyethyl)piperazine-1 -ethanesulfonic acid (HEPES) buffer using antibody concentration measurement with nanodrop (NANODROP 2000, Thermo Fisher Scientific, Waltham, MA, USA). To determine the number of chelates per antibody, MALDI mass spectrometry (KBSI, Ohchang, Korea) was performed. Matrix-assisted laser desorption ionization/time of flight (MALDI/TOF) mass spectra were obtained on an Ultraflextreme (Bruker Daltonics, Bremen, Germany) mass spectrometer using sinapinic acid as a matrix (Bruker Daltonics, Bremen, Germany).

^89^Zr was produced by MC-50 cyclotron (Scanditronix, Uppsala, Sweden) at the Korea Institute of Radiological and Medical Sciences (KIRAMS) and domestic RFT-30 cyclotron at the Korea Atomic Energy Research Institute (KAERI) via the ^89^Y(p,n)^89^Zr nuclear reaction and was finally obtained in the form of ^89^Zr-chloride. The ^89^Zr-chloride solution was argon purged, dried, and reconstituted with a 1 M HEPES buffer. ^89^Zr-chloride (74 MBq) was added to DFO-pertuzumab (2 mg/mL) solution. The reaction was performed at 37 °C for 60 min in a ThermoMixer^®^ (Eppendorf, Hamburg, Germany). The final solution was diluted with saline and filtered with a 0.22 μm syringe filter (Millex^®^GV, Millopore, Burlington, NJ, USA) for further experiments. The radiolabeling yield and radiochemical purity were determined by an instant thin layer chromatography-silica gel (ITLC-sg) and size exclusion-HPLC analysis, respectively. ITLC-sg analysis was performed by a Bioscan AR-2000 radio-TLC plate reader (Bioscan Inc., Washington, DC, USA) with ITLC-sg paper (Agilent Technologies, Forest Lakes, AZ, USA) as the stationary phase and 20 mM citrate buffer with 50 mM EDTA (pH 5.0) as the mobile phase. ^89^Zr-DFO-pertuzumab remained at the origin (*Rf* = 0), whereas free ^89^Zr migrated with the solvent front (*Rf* = 1). Size exclusion-HPLC was analyzed using a MAbPac SEC-1 column (Thermo Fisher Scientific, Waltham, MA, USA). The mobile phase consisted of 0.3 M NaCl in a 50 mM sodium phosphate buffer, pH 6.8, eluted at a flow rate of 0.2 mL/min. The retention time of radioimmunoconjugate was analyzed with UV absorbance (Younglin Instrument, Anyang, Korea) and radioactivity (GABI RI detector, Raytest, Angleur, Germany) detectors.

### 2.4. Affinity Test

The dissociation constant (K_d_) for ^89^Zr-DFO-pertuzumab was measured using radiolabeled pertuzumab binding to human HER2 antigen (Sino Biological Inc., Houston, TX, USA) coated 96-well plate with increasing the concentration of ^89^Zr-DFO-pertuzumab. Nonspecific binding was determined in presence of 100-fold molar excess of unlabeled pertuzumab. The K_d_ was calculated by fitting a plot of added ^89^Zr-DFO-pertuzumab (nM) versus the concentration of bound ^89^Zr-DFO-pertuzumab (nM) to a one-site saturation binding model using Prism^®^ Ver. 5.0 software (GraphPad Software, San Diego, CA, USA).

### 2.5. In Vitro Cell Binding Assay

To evaluate the HER2 expression level using ^89^Zr radiolabeled pertuzumab in breast cancer cell lines, in vitro cell binding assay was done. ^89^Zr radiolabeled pertuzumab (100 ng) was added to 1 × 10^6^ of breast cancer cells at 4 °C for 1 h. To determine whether pertuzumab binding to HER2 was inhibited by pretreatment of trastuzumab and herzuma, trastuzumab biosimilar, or not, trastuzumab and herzuma (10 μg) pretreated in JIMT-1 cells for 1 h at 4 °C and ^89^Zr radiolabeled pertuzumab (100 ng) was added at 4 °C for 1 h. Nonspecific binding was determined in the presence of 100-fold excess of pertuzumab. After incubation, the samples were washed twice in cold PBS containing 1% BSA. Each tube was counted in a gamma counter (WIZARD 1480, Perkin–Elmer, Waltham, MA, USA). Cell-bound radioactivity (%) was calculated by (cell-bound radioactivity—nonspecific binding radioactivity)/total radioactivity × 100.

To evaluate the correlation of HER2 expression by various concentrations of 17-DMAG (Selleck Chemicals, Houston, TX, USA) treatments, correlation analysis between flow cytometry and a cell-binding assay was performed by Prism^®^ Ver. 5.0 software (GraphPad Software, San Diego, CA, USA).

### 2.6. In Vitro Serum Stability

In vitro serum stability of ^89^Zr radioimmunoconjugates was evaluated for up to 7 days. An equal volume of human serum and radioimmunoconjugate was mixed and incubated at 37 °C. At each time point, the antibody-bound radioactivity (%) of samples was determined by radio-ITLC analysis.

### 2.7. In Vivo Evaluation of HER2 Expression in Brest Cancer Models

#### 2.7.1. Animal Model

All animal experiments were done under a protocol approved by KIRAMS Institutional Animal Care and Use Committee (IACUC, kirams2019-0025, 7 May 2019, kirams2021-0104, 9 December 2021). Female, 6-weeks aged, athymic BALB/c mice (DooYeol Biotech, Seoul, Korea) were used in all experiments. A total of 1 × 10^7^ of JIMT-1 or 5 × 10^6^ of MDA-MB-231 cells were subcutaneously injected into the right flank of each mouse. Animal experiments were conducted when each tumor size reached 100~200 mm^3^ after tumor implantation. Tumor volume and body weight were monitored twice a week.

#### 2.7.2. Biodistribution

The biodistribution of ^89^Zr-DFO-pertuzumab (*n* = 3/time point) was evaluated in JIMT-1 or MDA-MB-231 tumor-bearing mice. Each mouse was intravenously injected with ^89^Zr-DFO-pertuzumab (1.6~1.8 MBq/50 μg/100 μL). Biodistribution was performed at 2 h, 1 day, 3 days, 5 days, and 7 days post-injection of ^89^Zr-DFO-pertuzumab. The blood was collected by cardiac puncture and organs and tissues were excised. Samples were weighed and the amount of radioactivity was assessed in a gamma counter. Data were represented as the percentage of the injected radioactivity dose per gram of tissue (%ID/g). The tumor-to-blood (T/B), tumor-to-muscle (T/M), and tumor-to-liver (T/L) ratios were also calculated.

#### 2.7.3. Immuno-PET Imaging

To evaluate in vivo HER2 expression level using ^89^Zr-DFO-pertuzumab, immuno-PET imaging was performed in JIMT-1 and MDA-MB-231 tumor-bearing mice (*n* = 3/time point). ^89^Zr-DFO-pertuzumab (1.6~1.8 MBq/50 μg/100 μL) were intravenously injected into the mice and static scans were acquired for 1 h at 1 day, 5 days, and 7 days post-injection using a small animal PET scanner (microPET R4, Concorde Microsystems, Knoxville, TN, USA). Quantitative data were expressed as the percentage of the injected radioactivity dose per gram of tissue (%ID/g), which is calculated as the activity in target tissues divided by the decay corrected administered radioactivity × 100. Image visualization was performed using the ASIPro display software (microPET, Concorde Microsystems, Knoxville, TN, USA).

### 2.8. Immunotherapy

#### 2.8.1. Treatment Protocol

When the tumor volume reached 100~200 mm^3^, mice (*n* = 4/group) were intravenously administered with trastuzumab or isotype antibody (10 mg/kg) twice per week for 4 weeks. Tumor volume was calculated by long diameter × (short diameter)^2^/2, and body weight was measured thrice a week.

#### 2.8.2. Immuno-PET Imaging

To evaluate the dynamics of HER2 expression level by treatment of trastuzumab using ^89^Zr-DFO-pertuzumab, immuno-PET imaging was performed in JIMT-1 tumor-bearing mice (*n* = 3). ^89^Zr-DFO-pertuzumab (1.6~1.8 MBq/50 μg/100 μL) were intravenously injected into the mice at 22-days post-treatment of trastuzumab or isotype antibody and static scans were acquired for 1 h at 7 days post-injection using a small animal PET scanner. Quantitative data were expressed as standardized uptake values (SUV) [32]. Image visualization was performed using the ASIPro display software (microPET, Concorde Microsystems, Knoxville, TN, USA).

### 2.9. Heat Shock Protein 90 Inhibitor Treatment

#### 2.9.1. Treatment Protocol

When JIMT-1 tumor volume reached about 100~200 mm^3^, mice (*n* = 5/group) were intraperitoneally injected with heat shock protein 90 inhibitor (17-DMAG, Selleck Chemicals, Houston, TX, USA). Mice were administered a total of 150 mg/kg of 17-DMAG dissolved in 10% DMSO and 10% ethanol over 24 h in three doses of 50 mg/kg each. The control (vehicle, *n* = 5/group) mice were injected with an equal amount of saline in 10% DMSO and 10% ethanol. Tumor volume was calculated by long diameter × (short diameter)^2^/2, and body weight was measured thrice a week.

#### 2.9.2. Immuno-PET Imaging

To evaluate the pharmacodynamics of HER2 expression level by treatment of 17-DMAG, immuno-PET imaging was performed in JIMT-1 tumor-bearing mice. ^89^Zr-DFO-pertuzumab (1.6~1.8 MBq/50 μg/100 μL) were intravenously injected into the mice (control, *n* = 3; 17-DMAG, *n* = 5) at 7 days after 17-DMAG treatment and static scans were acquired for 1 h at 7 days post-injection using a small animal PET scanner. Quantitative data were expressed as a standardized uptake value (SUV). Image visualization was performed using the ASIPro display software.

#### 2.9.3. Western Blotting

At 7 days after 17-DMAG treatment, JIMT-1 tumors (Control, *n* = 3; 17-DMAG, *n* = 3) were collected and lysed in RIPA buffer (Thermo Fisher Scientific, Waltham, MA, USA) containing Halt protease and phosphatase inhibitors (Thermo Fisher Scientific, Waltham, MA, USA) for protein extraction. Protein lysates were separated using a 4–12% gradient SDS-polyacrylamide gel (Invitrogen, Waltham, MA, USA) and transferred to polyvinylidene difluoride membrane using an iBlot2 Dry Blotting System (Life Technologies, Carlsbad, CA, USA). After gel transfer, the membrane was incubated with 5% (*w*/*v*) skim milk in TBS-T solution for blocking, gently shaken for 2 h at room temperature, and then treated with an anti-HER2 primary antibody (Cell signaling, Danvers, MA, USA) overnight at 4 °C. Incubation of horseradish peroxidase-conjugated secondary antibody (Santa Cruz Biotechnology, Dallas, TX, USA) followed. After three additional washes in TBS-T, the immunoreactive bands were visualized using a SuperSignal West Pico PLUS Chemiluminescent Substrate (Thermo Fisher Scientific, Waltham, MA, USA). β-actin was used as the internal control.

#### 2.9.4. Immunohistochemistry

JIMT-1 tumors (control, *n* = 3; 17-DMAG, *n* = 3) were obtained at 7 days post-treatment and immediately fixed in 4% paraformaldehyde solution. Frozen embedded tissues were sectioned 4 μm-thick and subsequently stained. Tumor sections were immunostained with an anti-HER2 rabbit monoclonal antibody (1:100, Cell signaling, Danvers, MA, USA) and an FITC-conjugated goat anti-Rabbit IgG secondary antibody (1:100, Abcam, Cambridge, UK). Rat anti-mouse CD31 antibody (1:20, BD Korea, Seoul, Korea) and Cy3-conjugated AffiniPure goat anti-rat IgG (H + L) secondary antibody (1:100, Jackson Immunoresearch Laboratories, West Grove, PA, USA) were also used in the staining. Stained slides were mounted with VectaShield anti-fade mounting medium with DAPI (Vector Laboratories, Burlingame, CA, USA). The fluorescence images were obtained by a confocal laser scanning equipment, LSM 880 (Carl Zeiss Korea, Seoul, Korea), with a plan-apochromat 200× magnification using DAPI, FITC, and RFP filters and Zen 2.3 software (Trumbull, CT, USA).

### 2.10. Statistical Analysis

Quantitative data are represented as the mean ± SD. Statistical analysis was performed by Student’s t-test using Prism^®^ Ver. 5.0 software. *p* values of less than 0.05 were considered statistically significant.

## 3. Results

### 3.1. Preparation and Characterization of ^89^Zr-DFO-Pertuzumab

The average number of chelates per pertuzumab was determined to be 1.61 ± 0.02 by MALDI mass spectroscopy (Supplementary Figure S1). ^89^Zr-DFO-pertuzumab was successfully prepared at high radiolabeling yield and radiochemical purity (>98%) evaluated by ITLC-sg and size exclusion HPLC analysis (Supplementary Figures S2 and S3). ^89^Zr-DFO-pertuzumab showed favorable serum stability as >94% at 37 °C for 7 days (Supplementary Figure S4A). The affinity of ^89^Zr-DFO-pertuzumab to HER2 was measured with human HER2 antigen (250 ng). The dissociation constant (K_d_) for ^89^Zr-DFO-pertuzumab was 2.2 ± 0.4 nM (Supplementary Figure S4B).

### 3.2. Evaluation of HER2 Expression Level in Breast Cancer Cells

**Figure 1:**
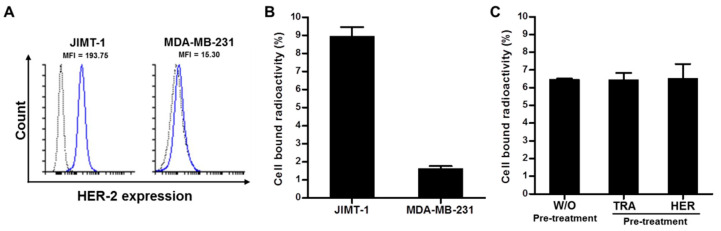
Analysis of HER2 expression level in breast cancer cells by flow cytometry and in vitro cell binding assay. (**A**) Flow cytometry in HER2-positive (JIMT-1) and negative (MDA-MB-231) breast cancer cells with pertuzumab. Gray-lined curve, isotype control; blue-lined curve, pertuzumab. (**B**) In vitro cell binding assay of ^89^Zr-DFO-pertuzumab in breast cancer cells. (**C**) In vitro cell binding assay of ^89^Zr-DFO-pertuzumab in JIMT-1 breast cancer cell by with or without (w/o) pre-treatment of trastuzumab (TRA) and herzuma (HER).

The expression level of HER2 was evaluated by flow cytometry using pertuzumab. JIMT-1 cells represented higher mean fluorescent intensity (MFI, 193.75) than MDA-MB-231 cells (MFI, 15.30) (Figure 1A). To determine the cell-surface HER2 expression using ^89^Zr-DFO-pertuzumab, a cell-binding assay was performed. The HER2 expression level of JIMT-1 cells was 8.95 ± 0.51%, while MDA-MB-231 cells was 1.62 ± 0.15% (Figure 1B). These results indicate that the binding of ^89^Zr-DFO-pertuzumab represents the level of HER2 expression in the cells. To determine whether pertuzumab binding to HER2 was inhibited by pretreatment of trastuzumab and herzuma or not, a cell-binding assay was performed after trastuzumab and herzuma treatment. The cell-bound radioactivity (%) of ^89^Zr-DFO-pertuzumab showed similar results with and without pretreatment of trastuzumab and herzuma. The binding of ^89^Zr-DFO-pertuzumab in the pretreatment of trastuzumab was 6.43 ± 0.04%, and that in the pretreatment of herzuma, trastuzumab biosimilar, was 6.52 ± 0.81%, compared to without pretreatment of HER2 antibody (6.47 ± 0.04%) (Figure 1C).

### 3.3. 17-DMAG Induces HER2 Degradation in JIMT-1 Cells In Vitro

**Figure 2:**
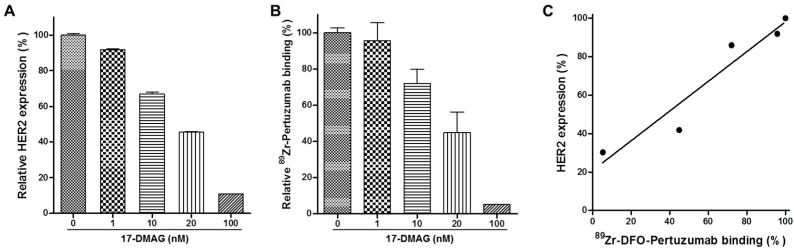
Pharmacodynamic change of HER expression level by treatment of heat shock protein 90 inhibitor in JIMT-1 breast cancer cells. (**A**) Flow cytometric analysis of HER2 expression of JIMT-1. Pertuzumab used as the primary antibody and monoclonal anti-human FITC-conjugated IgG antibody as the secondary antibody. (**B**) In vitro cell binding assay of ^89^Zr-DFO-pertuzumab. JIMT-1 cells were treated with various concentrations of heat shock protein 90 inhibitor (17-DMAG) for 24 h. (**C**) Correlation between flow cytometry analysis and cell binding assay. Correlation (r) = 0.9596.

17-DMAG-induced HER2 downregulation is shown in Figure 2A, B. The relative expression level of HER2 was evaluated by flow cytometry and cell binding assay. Relative HER2 expression (%) by treatment of 0, 1, 10, 20, 100 nM 17-DMAG for 24 h was 100.00 ± 0.81%, 91.84 ± 0.35%, 66.94 ± 1.13%, 45.53 ± 0.28% and 10.94 ± 0.08%, respectively (Figure 2A). Relative ^89^Zr-DFO-pertuzumab binding (%) by treatment of 0, 1, 10, 20, 100 nM 17-DMAG for 24 h was 100.00 ± 2.68%, 95.75 ± 9.81%, 72.07 ± 7.71%, 44.92 ± 11.41% and 5.27%, respectively (Figure 2B). These results suggest that 17-DMAG treatment induces HER2 downregulation in the cell surface of JIMT-1 tumor cells. The cell-bound radioactivity (%) of ^89^Zr-DFO-pertuzumab showed a similar pattern, with the HER2 expression (%) by flow cytometry. There was a clear correlation (r = 0.9596) between the cell-bound radioactivity (%) of ^89^Zr-DFO-pertuzumab and HER2 expression (%) by flow cytometry analysis (Figure 2C).

### 3.4. Biodistribution and Immuno-PET Imaging of ^89^Zr-DFO-Pertuzumab in Breast Cancer Xenograft Models

**Figure 3:**
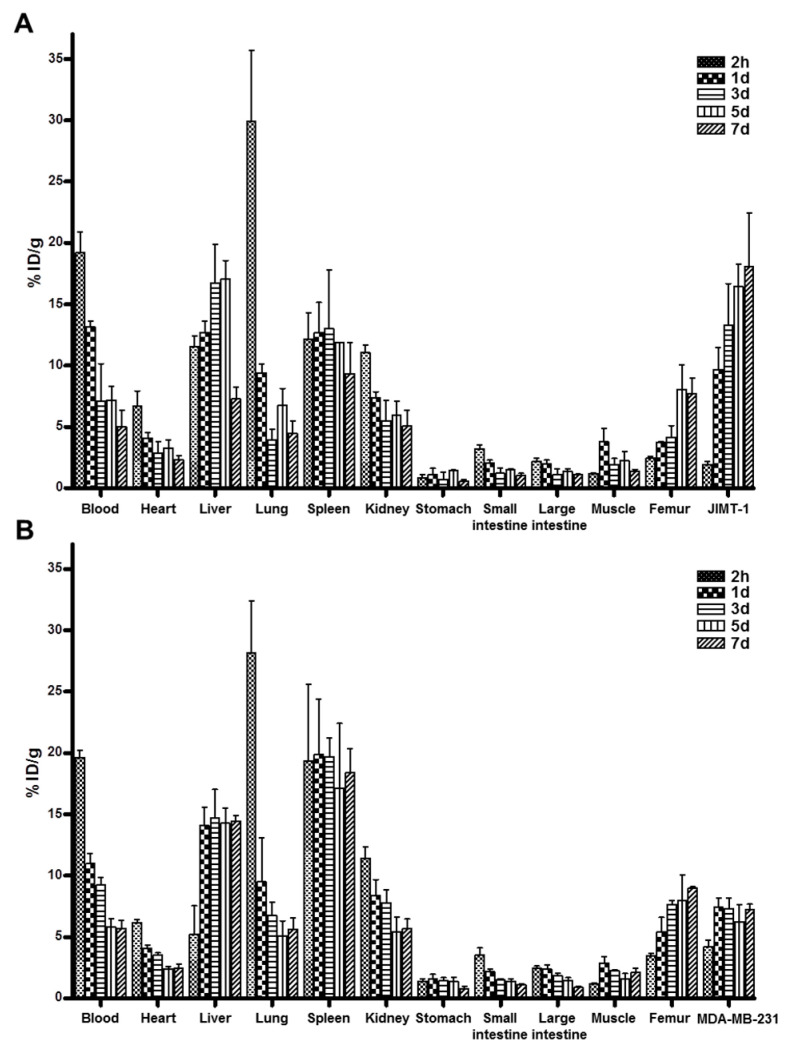
Biodistribution of ^89^Zr-DFO-pertuzumab in HER2-positive (JIMT-1) and -negative (MDA-MB-231) breast cancer xenograft models. ^89^Zr-DFO-pertuzumab (1.6~1.8 MBq/50 μg/100 μL) was injected intravenously into (**A**) HER2-positive (JIMT-1) and (**B**) negative (MDA-MB-231) breast cancer xenograft models. Biodistribution was performed at 2 h, 1 day, 3 days, 5 days, and 7 days post-injection of ^89^Zr-DFO-pertuzumab, and the amount of radioactivity of collected blood, organs, and tumor represented as percentage of the injected radioactivity dose/gram (%ID/g) was determined. Data were presented as mean ± sd (*n* = 3).

Biodistribution data of ^89^Zr-DFO-pertuzumab (*n* = 3/time point) are presented in Figure 3A, B. The biodistribution was performed at 2 h, 1 d, 3 days, 5 days, and 7 days post-injection of ^89^Zr-DFO-pertuzumab (1.6~1.8 MBq/50 μg/100 μL). In JIMT-1 xenograft models, the radioactivity of ^89^Zr-DFO-pertuzumab in blood was highest at 2 h with 19.22 ± 1.69 %ID/g and gradually decreased to 5.01 ± 1.39 %ID/g at 7 days post-injection. The radioactivity of tumors increased in a time-dependent manner. JIMT-1 tumor uptake of ^89^Zr-DFO-pertuzumab was 9.67 ± 1.79 %ID/g at 1 d and peaked at 7 days, reaching 18.06 ± 4.37 %ID/g. JIMT-1 tumor to blood (T/B), tumor to muscle (T/M), and tumor to liver (T/L) ratios at 1 d were 0.74 ± 0.15, 2.73 ± 1.13, and 0.76 ± 0.11, respectively. JIMT-1 tumor to blood (T/B), tumor to muscle (T/M), and tumor to liver (T/L) ratios at 7 days were 3.68 ± 0.61, 13.08 ± 2.26, and 2.47 ± 0.41, respectively. The T/B, T/M, and T/L of JIMT-1 tumors were markedly increased in a time-dependent manner. In MDA-MB-231 xenograft models, the radioactivity of ^89^Zr-DFO-pertuzumab in blood was highest at 2 h with 19.63 ± 0.57 %ID/g and gradually decreased to 5.7 ± 0.65 %ID/g at 7 days post-injection. MDA-MB-231 tumor uptake of ^89^Zr-DFO-pertuzumab peaked at 7.43 ± 0.74 %ID/g at 1 d and maintained with 7.22 ± 0.46 %ID/g at 7 days post-injection. MDA-MB-231 tumor to blood (T/B), tumor to muscle (T/M), and tumor to liver (T/L) ratios at 1 d were 0.68 ± 0.05, 2.61 ± 0.30, and 0.53 ± 0.06, respectively. MDA-MB-231 tumor to blood (T/B), tumor to muscle (T/M), and tumor to liver (T/L) ratios at 7 days were 1.27 ± 0.09, 3.47 ± 0.6, and 0.5 ± 0.05, respectively. The T/B and T/M of MDA-MB-231 tumors were slightly increased in a time-dependent manner. The T/L ratios of MDA-MB-231 tumors were maintained at all time points.

In biodistribution at 2 h, the lung and spleen showed high uptake in JIMT-1 and MDA-MB-231 tumor models. This result assumes that radiolabeled antibody aggregates were generated in the preparation, but on the other hand, the HPLC profiles (Supplementary Figure S3) presented correct radiochemical purity of the radiopharmaceutical and the lung and spleen uptakes in JIMT-1, and MDA-MB-231 tumor models gradually decreased in a time-dependent manner. Therefore, these results suggest that aggregates of ^89^Zr-DFO-pertuzumab do not exist in blood at 2 h.

The tumor uptake between MDA-MB-231 and JIMT-1 tumors showed a statistically significant difference from 1 day to 7 days. At 7 days post-injection, JIMT-1 tumor uptake was markedly higher than MDA-MB-231 tumor uptake.

**Figure 4:**
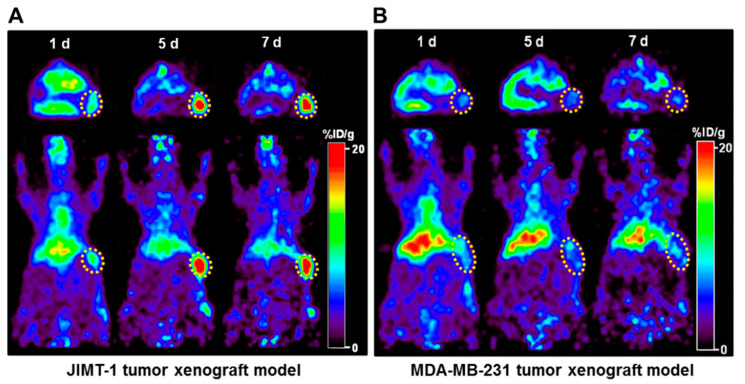
Immuno-PET imaging of ^89^Zr-DFO-pertuzumab in HER2-positive (JIMT-1) and -negative (MDA-MB-231) breast cancer xenograft models. PET images were acquired at 1 day, 5 days, and 7 days after injection of ^89^Zr-DFO-pertuzumab in (**A**) HER2-positive (JIMT-1) and (**B**) -negative (MDA-MB-231) tumors. Tumors are indicated as yellow dotted circles. The tumor uptake of ^89^Zr-DFO-pertuzumab was similar to that of the liver on day 1, but JIMT-1 tumor uptake increased, and liver uptake decreased in a time-dependent manner. At the same time, the uptake in the MDA-MB-231 tumor was relatively lower than that in the liver as time elapsed. All images were represented as the percentage of the injected radioactivity dose per gram of tissue (%ID/g).

To evaluate the potential of ^89^Zr-DFO-pertuzumab as an immuno-PET imaging agent for determining in vivo HER2 levels, we performed immuno-PET imaging in JIMT-1 (HER2-positive) and MDA-MB-231 (HER2-negative) xenograft models. Figure 4A,B show the transversal and coronal images of a representative mouse scanned at 1, 5, and 7 days post-injection of ^89^Zr-DFO-pertuzumab. At 7 days post-injection, JIMT-1 tumor uptake of ^89^Zr-DFO-pertuzumab was greatly higher than MDA-MB-231 tumor uptake at 7 days post-injection. These results suggest that ^89^Zr-DFO-pertuzumab as an immuno-PET imaging agent has the potential role for noninvasive and quantitative in vivo imaging of HER2 expression in breast cancer xenografted models.

### 3.5. Therapeutic Effects of Trastuzumab in the JIMT-1 Xenograft Model

**Figure 5:**
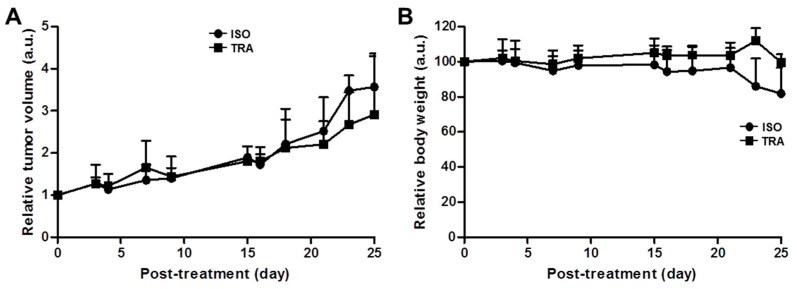
Therapeutic efficacy of trastuzumab in JIMT-1 xenograft model. (**A**) The tumor volumes were measured after injection of rituximab (isotype, ISO) and trastuzumab (TRA) with 10 mg/kg, twice per week for 4 weeks. Tumor volume was calculated by long diameter × (short diameter)^2^/2, and (**B**) Body weight was measured thrice a week. No apparent body weight loss was observed in all groups during treatment.

Anti-tumor effects of trastuzumab were assessed in the JIMT-1 xenograft model (Figure 5A,B). The growth rate of JIMT-1 tumors was similar between the isotype-treated group (ISO) and trastuzumab-treated group (TRA). There was no difference in tumor volume between ISO and TRA treatment in the JIMT-1 xenograft model (Figure 5A). TRA treatment was well tolerated in the JIMT-1 xenograft model, no apparent body weight loss was observed in all groups during treatment. (Figure 5B). These data suggest that the JIMT-1 tumor is resistant to trastuzumab immunotherapy.

### 3.6. Therapeutic Response Monitoring Using Immuno-PET Imaging in Trastuzumab-Treated JIMT-1 Tumors

**Figure 6:**
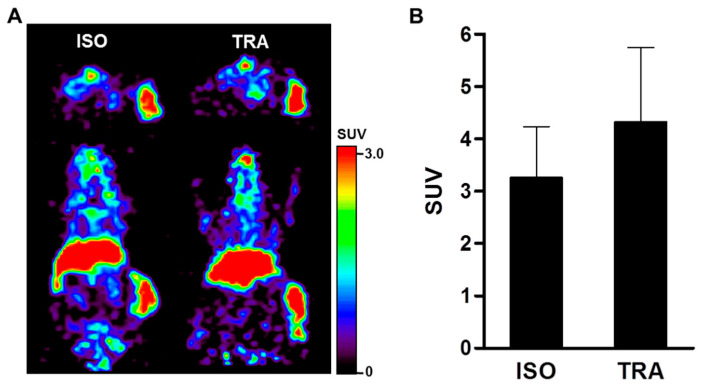
Immuno-PET imaging of ^89^Zr-DFO-pertuzumab by trastuzumab treatment in JIMT-1 breast cancer xenograft model. (**A**) To evaluate the dynamics of HER2 expression level by treatment of isotype (ISO) and trastuzumab (TRA) using ^89^Zr-DFO-pertuzumab, Immuno-PET imaging was performed in the JIMT-1 breast cancer xenograft model. Immuno-PET images were acquired 7 days after injection of ^89^Zr-DFO-pertuzumab. (**B**) JIMT-1 tumor uptake of ^89^Zr-DFO-pertuzumab as quantified using ASIPro display software. All images and quantitative data were expressed as a standardized uptake value (SUV).

To monitor therapeutic response by treatment of isotype (ISO) and trastuzumab (TRA) using the evaluation of HER2 expression level with ^89^Zr-DFO-pertuzumab, immuno-PET imaging was performed in the JIMT-1 breast cancer xenograft model. Immuno-PET imaging showed the transversal and coronal images of a representative mouse scanned at 7 days post-injection of ^89^Zr-DFO-pertuzumab. In both groups, PET images clearly showed the uptake of ^89^Zr-DFO-pertuzumab in JIMT-1 tumors at 7 days after injection (Figure 6A). The SUV of ^89^Zr-DFO-pertuzumab in JIMT-1 tumors showed statistically non-significant between ISO (3.27 ± 0.97) and TRA (4.33 ± 1.41) treated groups (*p* = 0.3432) (Figure 6B).

### 3.7. 17-DMAG Treatment Inhibits JIMT-1 Tumor Growth

**Figure 7:**
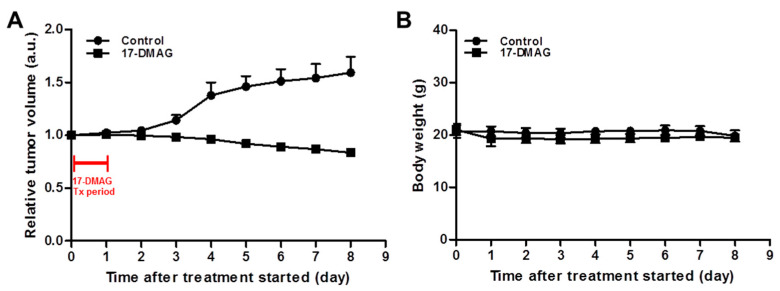
Therapeutic efficacy of heat shock protein 90 inhibitor (17-DMAG) in JIMT-1 xenograft model. (**A**) JIMT-1 tumor-bearing mice were treated with control (vehicle) or 17-DMAG. Mice were administered a total of 150 mg/kg of 17-DMAG over 24 h in three doses of 50 mg/kg each. The control mice were injected with an equal volume of the vehicle. (**B**) Body weight was measured thrice a week. No apparent body weight loss was observed in all groups during treatment.

The therapeutic efficacy of 17-DMAG in JIMT-1 tumors was evaluated and the relative tumor volume and body weight were shown in Figure 7. 17-DMAG is relatively nontoxic because of no significant difference in mouse body weight between the control (vehicle) and 17-DMAG-treated groups in JIMT-1 breast cancer xenograft model (Figure 7B). Mice were administered a total of 150 mg/kg of 17-DMAG over 24 h in three doses of 50 mg/kg each. The control mice were injected with an equal volume of vehicles. Relative tumor volumes in the control group increased in a time dependent manner, but those in the 17-DMAG-treated group were statistically significant and decreased to 84% of original tumor volumes (*p* < 0.0001).

### 3.8. Therapeutic Response Monitoring Using Immuno-PET Imaging in 17-DMAG-Treated JIMT-1 Tumors

**Figure 8:**
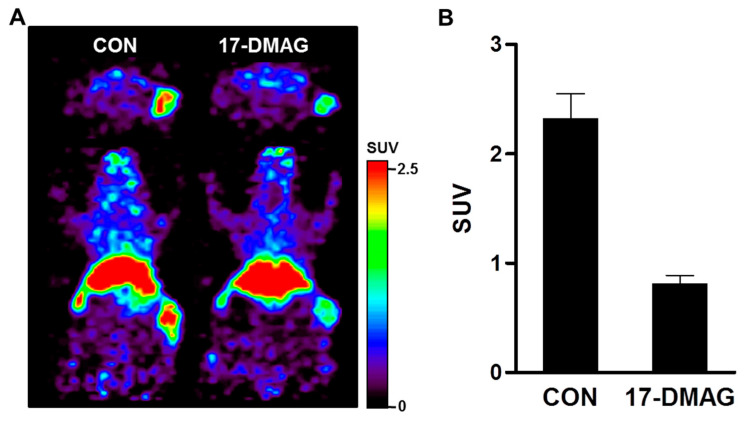
Immuno-PET imaging of ^89^Zr-DFO-pertuzumab by treatment of heat shock protein 90 inhibitor (17-DMAG) in JIMT-1 breast cancer xenograft model. (**A**) To evaluate the pharmacodynamics of HER2 expression level by treatment of 17-DMAG, Immuno-PET imaging was performed in the JIMT-1 breast cancer xenograft model. Mice were administered a total of 150 mg/kg of 17-DMAG over 24 h in three doses of 50 mg/kg each. PET images were acquired at 7 days post-injection of ^89^Zr-DFO-pertuzumab. Immuno-PET tracer uptake was markedly reduced by treatment of 17-DMAG (*p* < 0.0001). (**B**) JIMT-1 tumor uptake of ^89^Zr-DFO-pertuzumab as quantified using ASIPro display software. All images and quantitative data were expressed as the standardized uptake value (SUV).

To evaluate the pharmacodynamic change of HER2 after 17-DMAG treatment, ^89^Zr-DFO-pertuzumab immuno-PET scans were performed in the JIMT-1 breast cancer xenograft model. PET images were acquired at 7 days post-injection of ^89^Zr-DFO- pertuzumab. The tumor uptake of ^89^Zr-DFO-pertuzumab was markedly reduced by treatment of 17-DMAG (*p* < 0.0001) compared with the control group (CON) (Figure 8A). Quantitative data based on the SUV are shown in Figure 8B. In the CON group, the JIMT-1 tumor SUV of ^89^Zr-DFO-pertuzumab was 2.30 ± 0.23. In the 17-DMAG-treated group, JIMT-1 tumor SUV was significantly decreased to 0.80 ± 0.08 at 7 days post-injection (Figure 8B). There was no significant difference in ^89^Zr-DFO-pertuzumab uptake in other major organs between the 17-DMAG-treated and CON group. These results imply that HSP90 inhibitor, 17-DMAG, treatment causes HER2 downregulation in trastuzumab-resistant JIMT-1 tumors.

### 3.9. HER2 Expression Levels in 17-DMAG-Treated JIMT-1 Tumors

**Figure 9:**
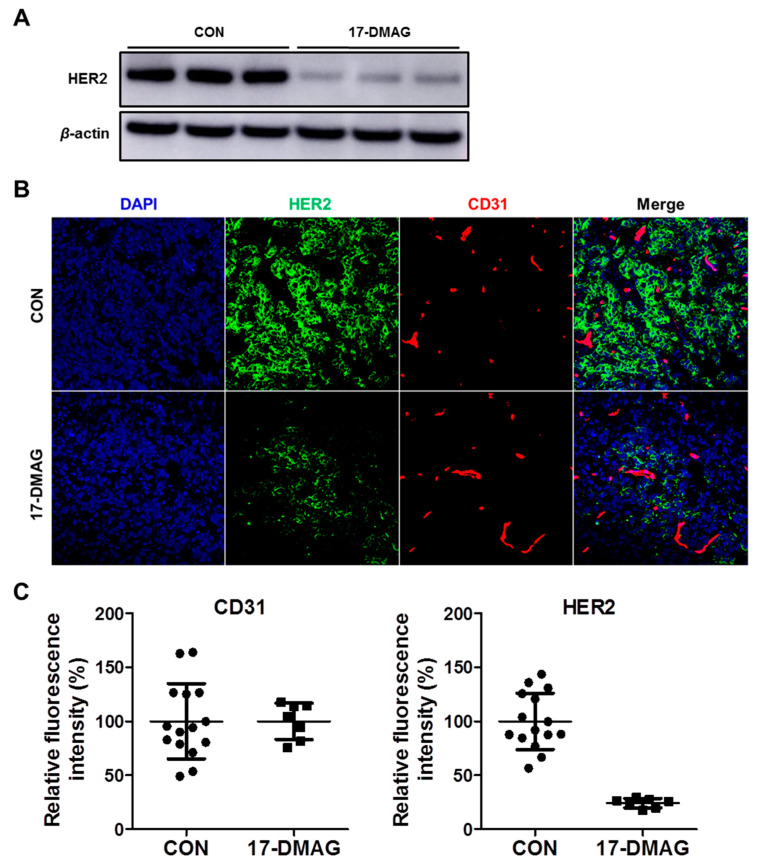
Western blot and immunofluorescence staining in JIMT-1 tumor treated with 17-DMAG. (**A**) Western blot analysis of HER2 expression in JIMT-1 tumor by treatment of 17-DMAG. JIMT-1 tumors were collected 7 days after 17-DMAG treatment. *β*-actin was used as an internal control. (**B**) Immunofluorescence staining of HER2 in control (CON, vehicle) and 17-DMA- treated JIMT-1 tumor tissues. Frozen section of JIMT-1 tumor was stained with CD31 (red), HER2 (green), and DAPI (blue). Images were acquired at 200× magnification. (**C**) Immunofluorescence staining of HER2 and CD31 was quantified using ImageJ software and quantitative data were expressed as relative fluorescence intensity (%). HER2 relative fluorescence intensity by 17-DMAG treatment was markedly reduced to 25% of control (*p* < 0.0001).

Additionally, to verify that 17-DMAG induces HER2 degradation in vivo, we performed western blot analysis and immunofluorescence staining. JIMT-1 tumors were collected 7 days after 17-DMAG treatment. Western blot analysis showed that HER2 expression level in 17-DMAG-treated JIMT-1 tumors markedly decreased compared to that in the CON group (Figure 9A). This result was consistent with ^89^Zr-DFO-pertuzumab PET imaging. Immunofluorescence staining was performed with anti-HER2 and anti-CD31 as primary antibodies and FITC-conjugated goat anti-Rabbit IgG and Cy3-conjugated AffiniPure goat anti-rat IgG (H + L), respectively, as the secondary antibody. Confocal fluorescence images were taken under the same conditions and scale to make sure that the relative brightness observed in the images reflected the difference in HER2 and CD31 expression levels. In the control JIMT-1 tumors (CON), HER-2 expression was very high as indicated by the strong pseudocolored green signal in tumors. After being treated with 17-DMAG, the HER-2 expression level was apparently low, due to a weak fluorescence signal (Figure 9B). HER2 relative fluorescence intensity (%) in the 17-DMAG treatment group was markedly reduced to 25% of the control group (*p* < 0.0001). There was no difference between CON and 17-DMAG-treated group in CD31 staining intensity (Figure 9C). Immuno-PET imaging showed reduced HER2 expression in 17-DMAG-treated JIMT-1 tumors, and western blot and immunofluorescence staining studies confirmed that HER2 expression level in JIMT-1 tumors was decreased by 17-DMAG treatment.

## 4. Discussion

In the present study, we describe the feasibility of antitumor efficacy monitoring using ^89^Zr-DFO-pertuzumab in human breast cancer xenograft models by immunotherapy, trastuzumab, HSP90 inhibitors, and 17-DMAG. ^89^Zr-DFO-pertuzumab was efficiently prepared at high radiolabeling yield and radiochemical purity (>98%) without further purification process. ^89^Zr-DFO-pertuzumab was used as an immuno-PET imaging agent for determining the HER2 expression level and differentiating between HER2-positive JIMT-1 tumors and HER2 negative MDA-MB-231 tumors in vivo. JIMT-1 tumor uptake of ^89^Zr-DFO-pertuzumab peaked at 7 days with 18.06 ± 4.37 %ID/g, compared to MDA-MB-231 tumor at 1 d with 7.22 ± 0.46 %ID/g (Figure 3). Immuno-PET imaging with ^89^Zr-DFO-pertuzumab determined therapeutic response in trastuzumab-resistant JIMT-1 tumors for refractory to trastuzumab treatment and responsive to 17-DMAG treatment, respectively.

In clinic, breast cancer patients with HER2 expression treated with pertuzumab in monotherapy demonstrated a poor response rate, but combination treatment with trastuzumab and chemotherapy resulted in augmented anticancer effect in patients [22]. Pertuzumab binds HER2 domain II and inhibits HER2 heterodimerization and trastuzumab binds HER2 domain IV and inhibits HER2 homodimerization; these represent a complementary mechanism of action of the two antibodies that could drive synergistic antitumor efficacy when given in combination strategy. Recently, FDA-approved antibody-drug conjugates, Ado-trastuzumab emtansine (Kadcyla^®^) and trastuzumab deruxtecan (Enhertu^®^) showed favorable therapeutic efficacy to HER2-positive breast cancer patients as trastuzumab-based drug conjugates. Therefore, pertuzumab could be useful to evaluate HER2 expression level as an immuno-PET imaging agent because it does not compete with trastuzumab binding to HER2 protein.

^89^Zr-DFO-pertuzumab was successfully prepared with high radiolabeling yield (>98%), radiochemical purity (>98%), in vitro serum stability with 94% at 7 days, specific activity with 5.48 GBq/μmole, and no further purification process. Additionally, the affinity of ^89^Zr-DFO-pertuzumab was 2.2 ± 0.4 nM. Marquez et al. reported that the affinity of ^89^Zr-pertuzumab was determined to be 2.4 ± 0.1 nM in SKBR3 cells [27]. Our affinity experiment was performed in solid-phase analysis with human HER2 protein and showed a similar affinity to the previous report.

In vitro ^89^Zr-DFO-pertuzumab cell-binding assay in JIMT-1 cells showed similar cell-bound radioactivity (%) regardless of the pretreatment of trastuzumab and herzuma (Figure 1C). In a previous study, Marquez et al. demonstrated that the in vitro binding of ^89^Zr-pertuzumab in BT-474 cells was increased by 30% in the presence of trastuzumab. Additionally, ^89^Zr-pertuzumab specifically accumulated in a HER2-positive BT-474 tumor and its tumor uptake was enhanced by the presence of trastuzumab [27]. Moreover, Fuentes et al. study showed that the pertuzumab binding affinity towards the HER2 in silico was increased which HER2 conformational changes to occur upon trastuzumab binding [33]. JIMT-1 tumor uptake in biodistribution of ^89^Zr-DFO-pertuzumab (50 μg/mice) slightly enhanced by trastuzumab immunotherapy (ISO vs. TRA, 3.27 ± 0.97 vs. 4.33 ± 1.41). However, there was no statistical significance between ISO and TRA. Previous reports [27] showed that trastuzumab pretreatment greatly enhanced ^89^Zr-DFO-pertuzumab (12~16 μg/mice) uptake in BT-474 tumors. However, other reports [34] found similar tumor uptake between Cy5-pertuzumab (50 μg/mice) alone and Cy5-pertuzumab in combination with trastuzumab in the KPL-4 tumor model. These discrepancies between our data and previous reports may be caused by the differences in the cell types and HER2 expression level in cells or differences in experimental condition such as an increased injected mass of radiolabeled or optical probe labeled pertuzumab.

The biodistribution of ^89^Zr-DFO-pertuzumab in HER2-positive (JIMT-1) and -negative (MDA-MB-231) breast cancer xenograft models showed a considerable degree of bone uptake (Figure 3). This result also reported that ^89^Zr-DFO-pertuzumab biodistribution in BT-474 and MDA-MB-231 xenograft models showed bone uptake [27]. ^89^Zr labeled antibodies degraded and released free ^89^Zr from ^89^Zr-DFO antibodies, and free ^89^Zr uptake in bones because ^89^Zr is a bone-seeking radionuclide. Recently, with the aim of reducing these undesirable bone uptakes, several groups [35] have developed new chelators for the preparation of ^89^Zr labeled antibodies. However, ^89^Zr bone uptake was not particularly noticeable in clinical studies, only observed in preclinical studies [26,36].

Chang et al. [37] showed that 3.75 µg of ^89^Zr-trastuzumab was administered via intravenous tail vein injection in HER2-positive MDA-MB-435-HER2 and negative MDA-MB-435-vector tumor xenograft models. The liver uptake showed about 8 %ID/g at 1 day and maintained with about 8 %ID/g at 4 days in the HER2-positive MDA-MB-435 tumor model. In HER2 negative MDA-MB-435, the liver uptake also showed about 8 %ID/g at 1 day and maintained with about 9 %ID/g at 4 days. Additionally, Chekol et al. [38] demonstrate that the injection of 10 µg of ^89^Zr-DFO-nimotuzumab showed that the liver uptake peaked at 3 days and decreased at 7 days in EGFR positive DLD-1 tumor model. In a low EGFR expressing MDA-MB-453 tumor model, liver uptake showed about 2 %ID/g at 1 day and about 5 %ID/g at 7 days. The liver uptake in MDA-MB-453 was increased in a time-dependent manner. In our data, 50 µg of ^89^Zr-DFO-pertuzumab was injected in JIMT-1 and MDA-MB-231 tumor models. In the HER2-positive JIMT-1 tumor model, liver uptake of ^89^Zr-DFO-pertuzumab peaked at 5 days and decreased at 7 days. The radiolabeled antibody concentration in the blood was decreased as time elapsed and tumor uptake of ^89^Zr-DFO-pertuzumab was increased by binding and internalization to the target. Therefore, the physiological liver uptake was also decreased at 7 days. However, the liver uptake of ^89^Zr-DFO-pertuzumab in the HER2 negative MDA-MB-231 tumor model was maintained. The radiolabeled antibody concentration in the blood decreased and the MDA-MB-231 tumor is HER2-negative tumor, which does not have HER2 target protein, ^89^Zr-DFO-pertuzumab could not be bound and internalized in the MDA-MB-231 tumor and tumor uptake maintained at all time points. Since the concentration of the radiolabeled antibody was relatively high dose compared to previous reports [37,38], instead of tumor accumulation, the physiological liver uptake of ^89^Zr-DFO-pertuzumab was maintained at all time points rather than decreased in a time-dependent manner.

Trastuzumab immunotherapy did not inhibit the tumor growth in JIMT-1 tumor models (Figure 5A). This finding was consistent with a previous report by Tanner et al., trastuzumab-resistant patient-derived JIMT-1 cells were insensitive to trastuzumab in vitro and in vivo xenograft models [39]. Trastuzumab is the most widely used monoclonal antibody for HER2-positive breast cancer. However, not all patients with HER2 overexpression benefit from HER2-targeted therapy with trastuzumab by various resistance mechanisms. Several mechanisms for trastuzumab resistance have been identified in preclinical studies and breast cancer patient-derived tumors [40]. Among them, TNFα-induced mucin 4 (MUC4) expression elicits trastuzumab resistance in HER2-positive JIMT-1 breast cancer models [41,42].

Immunotherapy results in the JIMT-1 xenograft model showed that tumor growth pattern was similar between rituximab, isotype, treated control and trastuzumab-treated groups (Figure 5). Additionally, immuno-PET imaging visualized that ^89^Zr-DFO-pertuzumab tumor uptake between isotype and trastuzumab treatment was not different (Figure 6). These data were consistent with previously reported studies [41,42] and confirmed that JIMT-1 had trastuzumab resistance in our models.

Heat shock protein 90 (HSP90) is a 90 kDa molecular chaperon and has a primary role in cellular homeostasis not only in normal cells but also in cancer cells for maintaining the activity of a variety of oncoproteins [43,44]. Thus, HSP90 is a promising target for anti-tumor therapy, because one of the most well-defined HSP90 client proteins in breast cancer is the ERBB2/Her2. The first HSP90 inhibitor, 17-AAG, an analog of geldanamycin was conducted in several clinical phase I and II trials for the treatment of patients with solid tumors and HER2-positive breast cancers [23,44]. However, 17-AAG has some problems such as poor water solubility, hepatotoxicity, and short biological half-life for its clinical usage [45]. Zsebik et al. found that 17-AAG decreased cell proliferation by promoting apoptosis in a dose-dependent manner and HSP90 combination with trastuzumab is more effective in HER2 downregulation in JIMT-1 cells [19].

A second HSP90 inhibitor, 17-DMAG, is a semi-synthetic derivative of geldanamycin. 17-DMAG has high solubility, improved formulation, better bioavailability, and greater anti-tumor potency than 17-AAG [46]. These various superiorities make it a more promising clinical drug. 17-DMAG has been studied in the preclinic and gone into several phase I trials as a single drug, and in combination with other potent anticancer agents in various types of solid tumors [13].

The immuno-PET imaging studies with ^89^Zr-DFO-pertuzumab were carried out to evaluate HER2 expression levels by 17-DMAG treatment. ^89^Zr-DFO-pertuzumab was specifically accumulated in isotype control groups and markedly reduced uptake in 17-DMAG-treated groups at 7 days post-injection (Figure 8A). Quantitative tumor SUVs of ^89^Zr-DFO-pertuzumab were significantly lower in 17-DMAG-treated groups (0.80 ± 0.08 SUV) than in vehicle control groups (2.30 ± 0.23 SUV) at 7 days post-injection (Figure 8B). Furthermore, the in vivo HER2 expression level was verified by western blot analysis. Western blot analysis showed that decreased HER2 protein level by 17-DMAG treatment (Figure 9A). Taken together, the effects of 17-DMAG in the JIMT-1 breast cancer model, 17-DMAG could become a useful therapeutic drug in the treatment of trastuzumab-resistant HER2 expressing tumors.

Our study showed the feasibility of therapeutic response monitoring with ^89^Zr-DFO-pertuzumab in trastuzumab-resistant and HSP90 inhibitor-sensitive JIMT-1 breast cancer tumor model. However, there was some limitation in our study. First, we conducted the experiment in an optimal single dose of the 17-DMAG showing the therapeutic effect, but it is necessary to evaluate the therapeutic response for treatment using various doses of 17-DMAG. This approach could be used to demonstrate the correlation between tumor progression and tumor uptake related to the HER2 expression level and whether there is a therapeutic effect with various doses of 17-DMAG treatment. Second, we elucidate the possibility of a therapeutic response monitoring with ^89^Zr-DFO-pertuzumab in a single optimal therapeutic dose of 17-DMAG at single immuno-PET imaging time point (7-days post-treatment). Long-term, repetitive therapeutic response monitoring with ^89^Zr-DFO-pertuzumab could manifestly demonstrate the HER2 dynamics of 17-DMAG treatment. However, repetitive ^89^Zr labeled antibody immuno-PET imaging could be performed at 3-week intervals for evaluating the dynamics of target molecules [31]. Further studies using long-term repetitive immuno-PET imaging at multiple time points are warranted to evaluate whether ^89^Zr-DFO-pertuzumab would be able to identify changes in HER2 dynamics with 17-DMAG treatment.

In previous reports, 17-DMAG inhibited fibroblast growth factor-2 induced angiogenesis. Kaur et al. reported that the HSP90 targeting agent, 17-DMAG, has a direct effect on endothelial cells through the inhibition of proliferation, migration, invasion, and induction of apoptosis [47]. However, immunofluorescence staining results showed that CD31 expression was not different between the vehicle and 17-DMAG treatment in JIMT-1 tumors (Figure 9B,C). These results implied that reduced tumor uptake of ^89^Zr-DFO-pertuzumab in 17-DMAG-treated groups was caused by not an antiangiogenic effect but HER2 downregulation of 17-DMAG treatment in JIMT-1 tumors. Despite the abundant achievements in the development of HSP90 inhibitors, none of these HSP90 inhibitors have successfully reached the market. Recently, to overcome these limitations, an N-terminal HSP90 inhibitor, NCT-547, was developed and showed the anti-tumor effect in trastuzumab-resistant HER2-positive breast cancer models [14].

In summary, our study highlights a new aspect of successful preclinical validation of immuno-PET imaging with ^89^Zr-DFO-pertuzumab for non-invasive and quantitative HER2 level monitoring for trastuzumab or 17-DMAG treatment. Additionally, immuno-PET imaging with ^89^Zr-DFO-pertuzumab selectively quantified to evaluate the HER2 downregulation of 17-DMAG treatment in trastuzumab-resistant JIMT-1 breast cancer models. Our results suggest that immuno-PET imaging using ^89^Zr-DFO-pertuzumab could be used to determine the target expression level and monitor the therapeutic response in HER2-positive cancer under various trastuzumab-based or HER2-targeted therapeutic regimens.

## 5. Conclusions

^89^Zr-DFO-pertuzumab specifically represents HER2 expression levels in HER2-positive and negative breast cancer models and non-invasively quantifies therapeutic responses for HER2-targeted treatments. This study will give insight into the usefulness of immuno-PET imaging that could be monitored for HER2 downregulation in trastuzumab-resistant breast cancers in clinical fields.

## Data Availability

The data are available within the article or from the corresponding author.

## Supplementary Materials

The following supporting information can be downloaded at: https://www.mdpi.com/article/10.3390/pharmaceutics14071338/s1, Figure S1: Determination of conjugated number of chelates per pertuzumab; Figure S2: Radiochromatograms of free ^89^Zr and ^89^Zr-DFO-pertuzumab; Figure S3: Size exclusion-HPLC chromatograms of pertuzumab, DFO-pertuzumab and ^89^Zr-DFO-pertuzumab; Figure S4: Human serum stability and affinity of ^89^Zr-DFO-pertuzumab.

## References

[1] Van Dongen G.A., Visser G.W., Lub-de Hooge M.N., de Vries E.G., Perk L.R. (2007). Immuno-PET: A navigator in monoclonal antibody development and applications. Oncologist.

[2] Smith-Jones P.M., Solit D.B., Akhurst T., Afroze F., Rosen N., Larson S.M. (2004). Imaging the pharmacodynamics of HER2 degradation in response to Hsp90 inhibitors. Nat. Biotechnol..

[3] Lamberts L.E., Williams S.P., van Scheltinga A.G., Lub-de Hooge M.N., Schröder C.P., Gietema J.A., Brouwers A.H., de Vries E.G. (2015). Antibody positron emission tomography imaging in anticancer drug development. J. Clin. Oncol..

[4] Song I.H., Lee T.S., Park Y.S., Lee J.S., Lee B.C., Moon B.S., An G.I., Lee H.W., Kim K.I., Lee Y.J. (2016). Immuno-PET Imaging and Radioimmunotherapy of ^64^Cu-/^177^Lu-Labeled Anti-EGFR Antibody in Esophageal Squamous Cell Carcinoma Model. J. Nucl. Med..

[5] Song I.H., Jeong M.S., Hong H.J., Shin J.I., Park Y.S., Woo S.K., Moon B.S., Kim K.I., Lee Y.J., Kang J.H. (2019). Development of a Theranostic Convergence Bioradiopharmaceutical for Immuno-PET based Radioimmunotherapy of L1CAM in Cholangiocarcinoma Model. Clin. Cancer Res..

[6] Wu A.M. (2009). Antibodies and antimatter: The resurgence of immuno-PET. J. Nucl. Med..

[7] Yarden Y., Sliwkowski M.X. (2001). Untangling the ErbB signalling network. Nat. Rev. Mol. Cell Biol..

[8] Shien T., Iwata H. (2020). Adjuvant and neoadjuvant therapy for breast cancer. Jpn. J. Clin. Oncol..

[9] Oh D.Y., Bang Y.J. (2020). HER2-targeted therapies—A role beyond breast cancer. Nat. Rev. Clin. Oncol..

[10] Verma S., Miles D., Gianni L., Krop I.E., Welslau M., Baselga J., Pegram M., Oh D.Y., Diéras V., Guardino E. (2012). Trastuzumab emtansine for HER2-positive advanced breast cancer. N. Engl. J. Med..

[11] Modi S., Saura C., Yamashita T., Park Y.H., Kim S.B., Tamura K., Andre F., Iwata H., Ito Y., Tsurutani J. (2020). Trastuzumab Deruxtecan in Previously Treated HER2-Positive Breast Cancer. N. Engl. J. Med..

[12] Ocaña A., Amir E., Pandiella A. (2020). HER2 heterogeneity and resistance to anti-HER2 antibody-drug conjugates. Breast Cancer Res..

[13] Mellatyar H., Talaei S., Pilehvar-Soltanahmadi Y., Barzegar A., Akbarzadeh A., Shahabi A., Barekati-Mowahed M., Zarghami N. (2018). Targeted cancer therapy through 17-DMAG as an Hsp90 inhibitor: Overview and current state of the art. Biomed. Pharmacother..

[14] Park J.M., Kim Y.J., Park S., Park M., Farrand L., Nguyen C.T., Ann J., Nam G., Park H.J., Lee J. (2020). A novel HSP90 inhibitor targeting the C-terminal domain attenuates trastuzumab resistance in HER2-positive breast cancer. Mol. Cancer.

[15] Sanchez J., Carter T.R., Cohen M.S., Blagg B.S.J. (2020). Old and New Approaches to Target the Hsp90 Chaperone. Curr. Cancer Drug Targets.

[16] Kamal A., Thao L., Sensintaffar J., Zhang L., Boehm M.F., Fritz L.C., Burrows F.J. (2003). A high-affinity conformation of Hsp90 confers tumour selectivity on Hsp90 inhibitors. Nature.

[17] Citri A., Alroy I., Lavi S., Rubin C., Xu W., Grammatikakis N., Patterson C., Neckers L., Fry D.W., Yarden Y. (2002). Drug-induced ubiquitylation and degradation of ErbB receptor tyrosine kinases: Implications for cancer therapy. EMBO J..

[18] Chiosis G., Timaul M.N., Lucas B., Munster P.N., Zheng F.F., Sepp-Lorenzino L., Rosen N. (2001). A small molecule designed to bind to the adenine nucleotide pocket of Hsp90 causes Her2 degradation and the growth arrest and differentiation of breast cancer cells. Chem. Biol..

[19] Zsebik B., Citri A., Isola J., Yarden Y., Szöllosi J., Vereb G. (2006). Hsp90 inhibitor 17-AAG reduces ErbB2 levels and inhibits proliferation of the trastuzumab resistant breast tumor cell line JIMT-1. Immunol. Lett..

[20] Modi S., Stopeck A.T., Gordon M.S., Mendelson D., Solit D.B., Bagatell R., Ma W., Wheler J., Rosen N., Norton L. (2007). Combination of trastuzumab and tanespimycin (17-AAG, KOS-953) is safe and active in trastuzumab-refractory HER-2 overexpressing breast cancer: A phase I dose-escalation study. J. Clin. Oncol..

[21] Metzger-Filho O., Winer E.P., Krop I. (2013). Pertuzumab: Optimizing HER2 blockade. Clin. Cancer Res..

[22] Cortés J., Fumoleau P., Bianchi G.V., Petrella T.M., Gelmon K., Pivot X., Verma S., Albanell J., Conte P., Lluch A. (2012). Pertuzumab monotherapy after trastuzumab-based treatment and subsequent reintroduction of trastuzumab: Activity and tolerability in patients with advanced human epidermal growth factor receptor 2-positive breast cancer. J. Clin. Oncol..

[23] Kim H.J., Park J.Y., Lee T.S., Song I.H., Cho Y.L., Chae J.R., Kang H., Lim J.H., Lee J.H., Kang W.J. (2019). PET imaging of HER2 expression with an 18F-fluoride labeled aptamer. PLoS ONE.

[24] Xu Y., Wang L., Pan D., Yu C., Mi B., Huang Q., Sheng J., Yan J., Wang X., Yang R. (2019). PET imaging of a ^68^Ga labeled modified HER2 affibody in breast cancers: From xenografts to patients. Br. J. Radiol..

[25] Qi S., Hoppmann S., Xu Y., Cheng Z. (2019). PET Imaging of HER2-Positive Tumors with Cu-64-Labeled Affibody Molecules. Mol. Imaging Biol..

[26] Dijkers E.C., Oude Munnink T.H., Kosterink J.G., Brouwers A.H., Jager P.L., de Jong J.R., van Dongen G.A., Schröder C.P., Lub-de Hooge M.N., de Vries E.G. (2010). Biodistribution of ^89^Zr-trastuzumab and PET imaging of HER2-positive lesions in patients with metastatic breast cancer. Clin. Pharmacol. Ther..

[27] Marquez B.V., Ikotun O.F., Zheleznyak A., Wright B., Hari-Raj A., Pierce R.A., Lapi S.E. (2014). Evaluation of ^89^Zr-pertuzumab in Breast cancer xenografts. Mol. Pharm..

[28] Ulaner G.A., Carrasquillo J.A., Riedl C.C., Yeh R., Hatzoglou V., Ross D.S., Jhaveri K., Chandarlapaty S., Hyman D.M., Zeglis B.M. (2020). Identification of HER2-Positive Metastases in Patients with HER2-Negative Primary Breast Cancer by Using HER2-targeted ^89^Zr-Pertuzumab PET/CT. Radiology.

[29] Niu G., Li Z., Cao Q., Chen X. (2009). Monitoring therapeutic response of human ovarian cancer to 17-DMAG by noninvasive PET imaging with ^64^Cu-DOTA-trastuzumab. Eur. J. Nucl. Med. Mol. Imaging.

[30] Munnink T.H., de Korte M.A., Nagengast W.B., Timmer-Bosscha H., Schröder C.P., de Jong J.R., Dongen G.A., Jensen M.R., Quadt C., Hooge M.N. (2010). ^89^Zr-trastuzumab PET visualises HER2 downregulation by the HSP90 inhibitor NVP-AUY922 in a human tumour xenograft. Eur. J. Cancer.

[31] Massicano A.V.F., Lee S., Crenshaw B.K., Aweda T.A., El Sayed R., Super I., Bose R., Marquez-Nostra B.V., Lapi S.E. (2019). Imaging of HER2 with [^89^Zr]pertuzumab in Response to T-DM1 Therapy. Cancer Biother. Radiopharm..

[32] Lee T.S., Song I.H., Shin J.I., Park Y.S., Kim J.Y., Kim K.I., Lee Y.J., Kang J.H. (2018). PET Imaging Biomarkers of Anti-EGFR Immunotherapy in Esophageal Squamous Cell Carcinoma Models. Cells.

[33] Fuentes G., Scaltriti M., Baselga J., Verma C.S. (2011). Synergy between trastuzumab and pertuzumab for human epidermal growth factor 2 (Her2) from colocalization: An in silico based mechanism. Breast Cancer Res..

[34] Scheuer W., Friess T., Burtscher H., Bossenmaier B., Endl J., Hasmann M. (2009). Strongly enhanced antitumor activity of trastuzumab and pertuzumab combination treatment on HER2-positive human xenograft tumor models. Cancer Res..

[35] Feiner I.V.J., Brandt M., Cowell J., Demuth T., Vugts D., Gasser G., Mindt T.L. (2021). The Race for Hydroxamate-Based Zirconium-89 Chelators. Cancers.

[36] Deri M.A., Zeglis B.M., Francesconi L.C., Lewis J.S. (2013). PET imaging with ⁸⁹Zr: From radiochemistry to the clinic. Nucl. Med. Biol..

[37] Chang A.J., Desilva R., Jain S., Lears K., Rogers B., Lapi S. (2012). ^89^Zr-Radiolabeled Trastuzumab Imaging in Orthotopic and Metastatic Breast Tumors. Pharmaceuticals.

[38] Chekol R., Solomon V.R., Alizadeh E., Bernhard W., Fisher D., Hill W., Barreto K., DeCoteau J.F., Parada A.C., Geyer C.R. (2018). ^89^Zr-nimotuzumab for immunoPET imaging of epidermal growth factor receptor I. Oncotarget.

[39] Tanner M., Kapanen A.I., Junttila T., Raheem O., Grenman S., Elo J., Elenius K., Isola J. (2004). Characterization of a novel cell line established from a patient with Herceptin-resistant breast cancer. Mol. Cancer Ther..

[40] Rexer B.N., Arteaga C.L. (2012). Intrinsic and acquired resistance to HER2-targeted therapies in HER2 gene-amplified breast cancer: Mechanisms and clinical implications. Crit. Rev. Oncog..

[41] Nagy P., Friedländer E., Tanner M., Kapanen A.I., Carraway K.L., Isola J., Jovin T.M. (2005). Decreased accessibility and lack of activation of ErbB2 in JIMT-1, a herceptin-resistant, MUC4-expressing breast cancer cell line. Cancer Res..

[42] Mercogliano M.F., De Martino M., Venturutti L., Rivas M.A., Proietti C.J., Inurrigarro G., Frahm I., Allemand D.H., Deza E.G., Ares S. (2017). TNFα-Induced Mucin 4 Expression Elicits Trastuzumab Resistance in HER2-Positive Breast Cancer. Clin. Cancer Res..

[43] Citri A., Kochupurakkal B.S., Yarden Y. (2004). The achilles heel of ErbB-2/HER2: Regulation by the Hsp90 chaperone machine and potential for pharmacological intervention. Cell Cycle.

[44] Banerji U., O’Donnell A., Scurr M., Pacey S., Stapleton S., Asad Y., Simmons L., Maloney A., Raynaud F., Campbell M. (2005). Phase I pharmacokinetic and pharmacodynamic study of 17-allylamino, 17-demethoxygeldanamycin in patients with advanced malignancies. J. Clin. Oncol..

[45] Garcia-Carbonero R., Carnero A., Paz-Ares L. (2013). Inhibition of HSP90 molecular chaperones: Moving into the clinic. Lancet Oncol..

[46] Smith V., Sausville E.A., Camalier R.F., Fiebig H.H., Burger A.M. (2005). Comparison of 17-dimethylaminoethylamino-17-demethoxy-geldanamycin (17DMAG) and 17-allylamino-17-demethoxygeldanamycin (17AAG) in vitro: Effects on Hsp90 and client proteins in melanoma models. Cancer Chemother. Pharmacol..

[47] Kaur G., Belotti D., Burger A.M., Fisher-Nielson K., Borsotti P., Riccardi E., Thillainathan J., Hollingshead M., Sausville E.A., Giavazzi R. (2004). Antiangiogenic properties of 17-(dimethylaminoethylamino)-17-demethoxygeldanamycin: An orally bioavailable heat shock protein 90 modulator. Clin. Cancer Res..

