# Supplementary material for: Therapeutic Response Monitoring with 89Zr-DFO-Pertuzumab in HER2-Positive and Trastuzumab-Resistant Breast Cancer Models
Source: Pharmaceutics. 2022 Jun 24;14(7):1338. doi: 10.3390/pharmaceutics14071338 (PMC9324044; doi:10.3390/pharmaceutics14071338)
Supplement: Supplementary file 1 [file pharmaceutics-14-01338-s001.zip › pharmaceutics-1745518-supplementary.pdf]

Supplementary File

Therapeutic Response Monitoring with ^{89}Zr-DFO-Pertuzumab in HER2 Positive and Trastuzumab Resistant Breast Cancer Model

Minwoo Kang [1,†], Jong Il Shin [1,†], Sangjin Han [1], Jung Young Kim [1], Jeonghoon Park [2], Kwang Il Kim [1], Joo Hyun Kang [1] and Tae Sup Lee [1,*]

[1]Division of RI Application, Korea Institute of Radiological and Medical Sciences (KIRAMS), 75 Nowon-ro, Nowon-gu, Seoul, 01812, South Korea;

[2]Korea Atomic Energy Research Institute (KAERI), 29 Geumgu-gil, Jeongup-si, Jeollabuk-do, 56212, South Korea;

[†]These authors equally contributed to this work.

[*]Correspondence: nobelcow@kirams.re.kr; Tel.: +82-2-970-1351

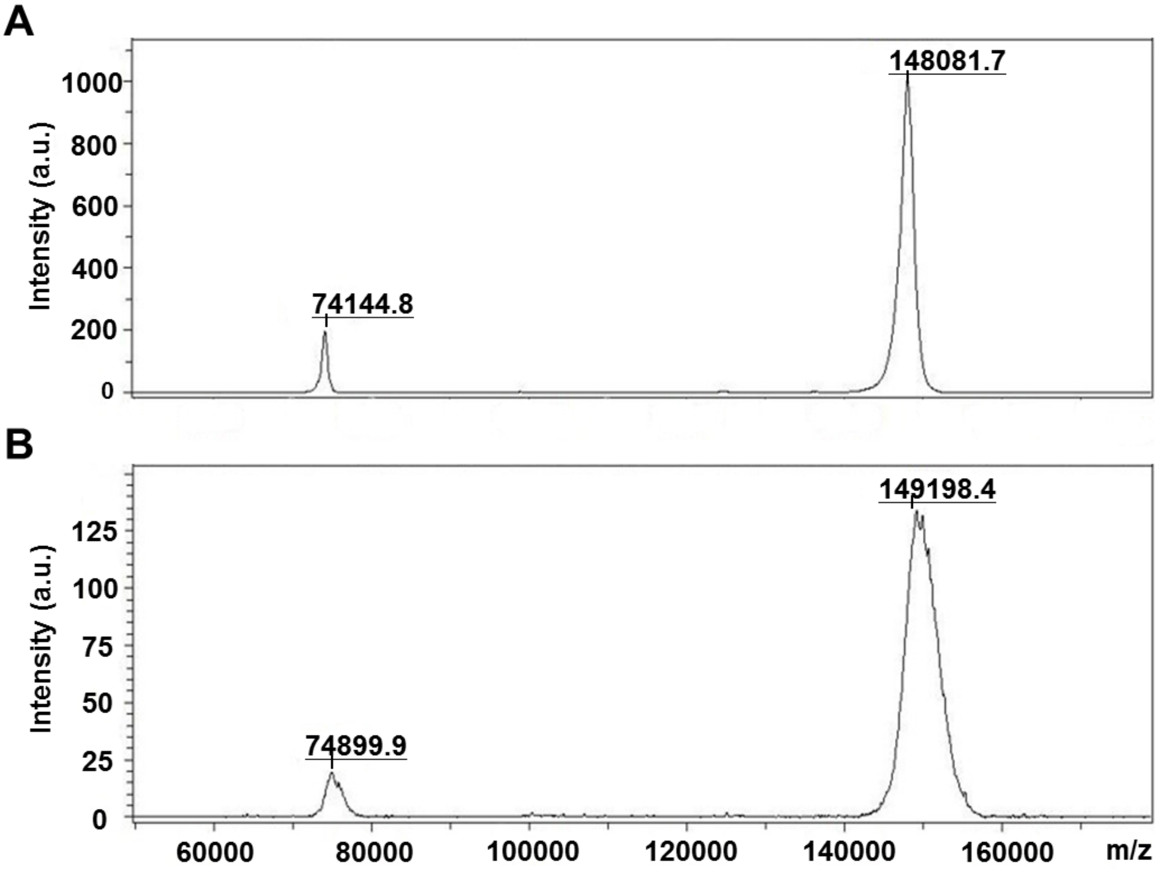

Supplementary Figure S1. Determination of conjugated number of chelates per pertuzumab. MALDI-TOF mass spectra of (A) pertuzumab and (B) DFO-pertuzumab. Pertuzumab was conjugated with DFO as molar ratio 1:10. The molecular peaks gives a degree of 1.61 ± 0.02 DFOs per pertuzumab. a.u., Arbitrary unit.

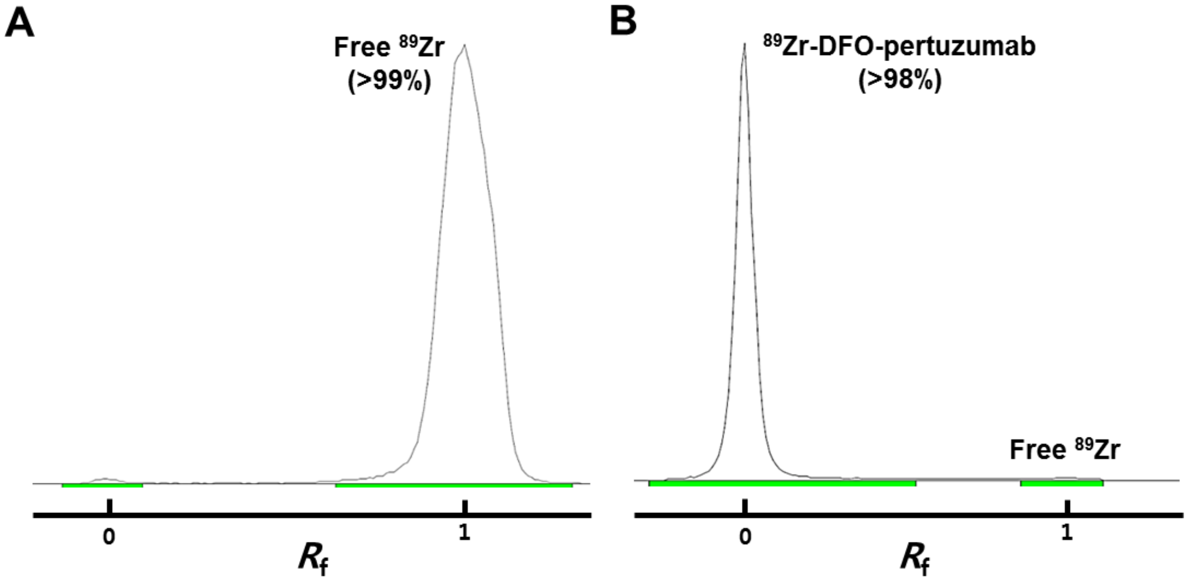

Supplementary Figure S2. Radiochromatograms of free ^{89}Zr and ^{89}Zr-DFO-pertuzumab.
Analysis of radiolabeling yield and radiochemical purity of ^{89}Zr-DFO-pertuzumab. Instant thin layer chromatography (ITLC) of free ^{89}Zr (A) and ^{89}Zr-DFO-pertuzumab (B). ITLC analysis was performed using ITLC-sg as stationary phase and 20 mM citrate buffer pH 5 with 50 mM EDTA as mobile phase. ^{89}Zr was successfully radiolabeled with DFO-pertuzumab at high radiolabeling yield and radiochemical purity (> 98%) without further purification.

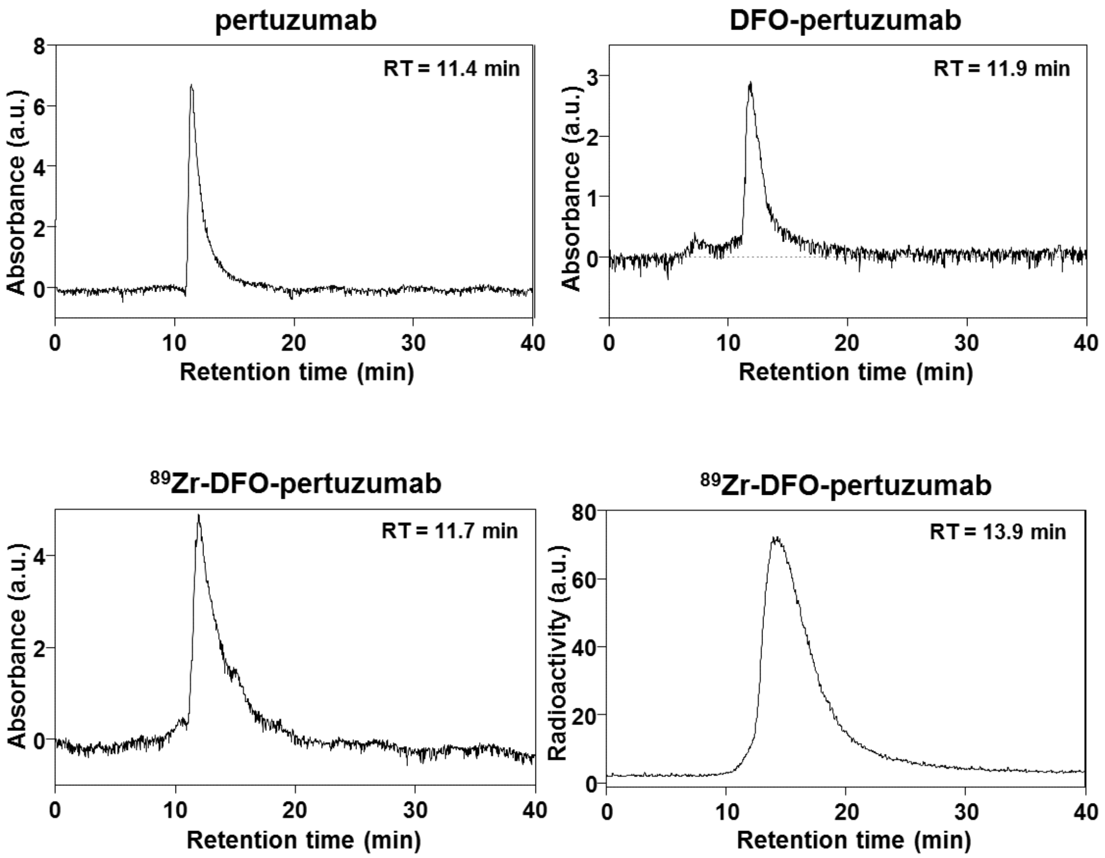

Supplementary Figure S3. Size exclusion-HPLC chromatograms of pertuzumab, DFO-pertuzumab and ^{89}Zr-DFO-pertuzumab. The purity was determined by HPLC analysis with MAbPac SEC-1 as a stationary phase and 0.3 M NaCl in 50 mM sodium phosphate buffer, pH 6.8 as a mobile phase. Samples were eluted at a flow rate of 0.2 mL/min. The retention time of pertuzumab, DFO-pertuzumab, ^{89}Zr-DFO-pertuzumab was determined by UV absorbance detector at 280 nm and GABI RI detector. The difference of retention time of ^{89}Zr-DFO-pertuzumab (11.7 min vs. 13.9 min) between absorbance and radioactivity detectors is due to the physical distance between absorbance and radiactivity detector. a.u., Arbitrary unit

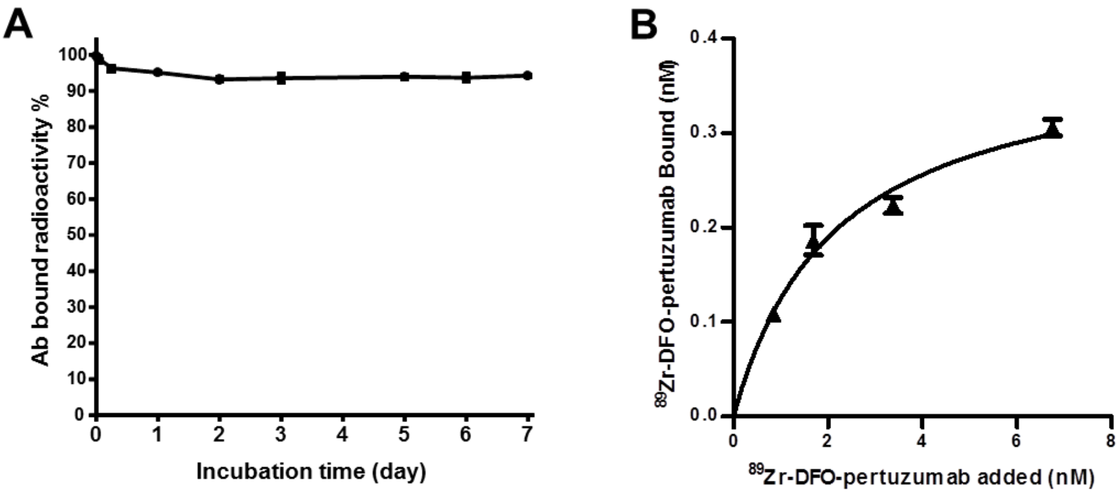

Supplementary Figure S4. Human serum stability and affinity of ^{89}Zr-DFO-pertuzumab.
(A) *In vitro* human serum stability of ^{89}Zr-DFO-pertuzumab. Radioimmunoconjugates were mixed with equal volume of human serum and incubated at 37℃. The antibody bound radioactivity (%) of samples was determined by ITLC analysis at each time point. ^{89}Zr-DFO-pertuzumab showed favorable serum stability with above 94% at 37℃ until 7 days. (B) The affinity of radiolabeled pertuzumab to HER2 measured with human HER2 antigen (250 ng) coated 96-well plate. Nonspecific binding was evaluated with pretreatment of 100-fold excess cold trastuzumab antibody. K_d value was analyzed with Prism 5. K_d value of ^{89}Zr-DFO-pertuzumab was 2.2 ± 0.4 nM.
